# Serum globulin and albumin to globulin ratio as potential diagnostic biomarkers for periprosthetic joint infection: a retrospective review

**DOI:** 10.1186/s13018-020-01959-1

**Published:** 2020-10-07

**Authors:** Yongyu Ye, Weishen Chen, Minghui Gu, Guoyan Xian, Baiqi Pan, Linli Zheng, Ziji Zhang, Puyi Sheng

**Affiliations:** Department of Orthopedic Surgery, The First Affiliated Hospital, Sun Yat-sen University, 58 Zhongshan 2nd Road, Guangzhou, 510080 China

**Keywords:** Periprosthetic joint infection, Globulin, Albumin to globulin ratio, Diagnosis

## Abstract

**Background:**

Periprosthetic joint infection (PJI) has been increasingly documented; however, its preoperative accurate diagnosis remains challenging. Furthermore, there is a dire need to identify appropriate and effective biomarkers. We aimed to evaluate the relationship between globulin, albumin to globulin (A/G) ratio, and development of PJI in patients undergoing revision total joint arthroplasty (TJA).

**Methods:**

A retrospective study was conducted on patients who had undergone revision TJA between 2011 and 2018 (89 with aseptic mechanic failure and 38 with PJI). The serum proteins were explored using univariate analysis followed by multivariate logistic regression. The diagnostic performance of these proteins was assessed by the receiver operating characteristic (ROC) curve.

**Results:**

Higher globulin levels (odds ratio [OR], 1.239; *P* < 0.001) and lower A/G ratio (OR, 0.007; *P* < 0.001) were strongly associated with the risk of PJI. ROC curve analysis demonstrated reasonable diagnostic performance for globulin (area under the curve [AUC], 0.77; sensitivity, 78.95%; and specificity, 69.66%) and A/G ratio (AUC, 0.779; sensitivity, 65.79%; and specificity, 78.65%).

**Conclusions:**

Both globulin and A/G ratio were associated with PJI and may serve as potential adjuvant biomarkers in the diagnosis of PJI.

## Background

Total joint arthroplasty (TJA) is a successful surgery to relieve pain and improve the quality of life. However, subsequent complications, especially the periprosthetic joint infection (PJI), have been increasingly recorded over decades [1–3]. The rate of debilitating PJI following TJA is approximately 1–2.5%, imposing a huge burden on the individuals and health care system [3, 4]. Furthermore, the variability in the clinical presentation and the bacterial profile as well as the lack of reliable biomarkers make the timely and accurate diagnosis of PJI even more challenging. Though several biomarkers have been developed as per the Musculoskeletal Infection Society (MSIS) guidelines recently [2, 5, 6], some cases still remain unidentified or misdiagnosed, particularly in those with chronic and low virulence infections [6, 7]. Consequently, additional efforts are required to optimize PJI diagnosis by determining the appropriate and effective biomarkers.

Inflammation and infection play a vital role in the process of developing PJI and various markers related to these processes such as the C-reactive protein (CRP) and erythrocyte sedimentation rate (ESR) have been investigated for their ability to predict PJI [8, 9]. Globulin, an easily accessible and reliable biomarker in the basic metabolic panel, has proven to be a critical marker associated with inflammation and infection [10]. Numerous studies have shown elevated serum globulin levels and decreased albumin to globulin (A/G) ratio in patients with infection and advanced malignancy, suggesting their important role in the immune and inflammatory system [11–16]. However, until now, no strong consensus focusing on the association between globulin, A/G ratio, and the development of PJI has been elucidated.

Therefore, we aimed to assess the association of globulin and A/G ratio with PJI and their ability to be used as diagnostic biomarkers.

## Materials and methods

### Study design and criteria of population

Following approval by our institutional review board, we retrospectively reviewed patients who had undergone revision total hip or knee arthroplasties from December 2011 to November 2018 in our institution. Additionally, another inclusion criterion was the availability of preoperative globulin and A/G ratio values. Our study was carried out in accordance with the relevant protocols and regulations. The requirement for informed consent was waived due to the retrospective design of our study. A total of 159 revision patients who met the inclusion requirement were initially identified. Unfortunately, 32 patients were excluded after applying the exclusion criteria. Patients experiencing other infective or inflammatory diseases, including tuberculosis (*n* = 1), rheumatoid arthritis (*n* = 3), ankylosing spondyloarthritis (*n* = 3), and superficial incisional wound infection (*n* = 5), were excluded. Moreover, patients with severe liver or renal dysfunction (*n* = 2) were excluded as serum proteins are largely produced in the liver and some are metabolized in the kidney [10]. Those who had undergone revision surgery secondary to dislocation (*n* = 12), breakage of the prosthesis (*n* = 1), and periprosthetic fracture (*n* = 5), were excluded because most of these complications might have been caused by violence rather than infection. This resulted in a cohort of 89 patients with aseptic mechanic failure and 38 patients with septic revision.

### Diagnostic criteria and management protocol

The clinical features of revision patients were comprehensively interpreted by the attending physician on admission. Preoperative standardized tests, including routine blood tests, chest radiographs, and electrocardiogram, were performed for all patients. After a thorough evaluation, additional tests such as the echocardiogram were ordered as necessary by the attending physician. The MSIS criteria were applied for the diagnosis of PJI [2, 6]. All revision patients with the suspicion of PJI were further investigated following the assessment protocol in our department. In patients with suspected PJI, dramatically increased preoperative serological biomarkers related to infection or positive imaging findings were observed prior to joint aspiration. Synovial fluid was sent to the lab for further analysis (color, clarity, white blood cell count, polymorphonuclear differential, Rivalta test, gram stain, and bacterial culture). Importantly, antibiotics were not routinely given preoperatively unless the physician had such evidence according to the sensitivity test of bacterial culture. Intraoperatively, cultures were sampled from different sites around the prosthesis. After that, diluted povidone-iodine irrigation was used for at least three times to eliminate any chances of contamination. For an accurate diagnosis, the surgeon obtainedbiopsy samples for histologic analysis. Intravenous antibiotics, namely second-generation cephalosporin, were routinely prescribed during surgery and were continued afterward until a positive result from the bacterial sensitivity test was noted. Then, the sensitive antibiotics were administered. All surgeries were performed by one of the five skilled arthroplasty surgeons. All knee revision procedures were done using a medial parapatellar approach while the hip surgeries were performed utilizing a posterior lateral approach. Unless contraindicated, non-steroidal anti-inflammatory drugs or anesthetic pump was used for patients and prophylactic heparin or other antithrombotic therapy was initiated within 24 h postoperatively and continued for nearly 1 month. Subsequently, standardized physical rehabilitation protocols were implemented for all patients. Postoperative routine blood tests (e.g., complete cell count and infection-related biomarkers) were regularly examined. Patients were subjected to regular follow-ups in the clinic after discharge.

### Data collection and laboratory examination

A comprehensive review of clinical information was performed retrospectively. Baseline demographic features, including age, sex, operative joint, smoking, alcohol, start and end time of operation, admission and discharge date, and date of surgery, were collected. Patients who complicated with diabetes mellitus, hypertension, and dysfunction of kidney or liver were identified. Moreover, in order to minimize the interference of globulin level and A/G ratio affected by other inflammatory and infective disorders, possible confounding diseases, including ankylosing spondylitis, rheumatoid arthritis, and tuberculosis were documented. Finally, the reasons for readmission were all carefully noted, including dislocation, periprosthetic fracture, breakage of prostheses, surgical site infection, and aseptic mechanic failure (malalignment, aseptic loosening, instability, aseptic wear, or other unexplained pain). Last but not least, preoperative serum protein values (total protein, albumin, globulin, and A/G ratio) were obtained from the basic metabolic panel drawn in the morning after patients underwent night-time fasting for a minimum of 7 h. All tests were performed as soon as possible by our laboratory with standardized protocols.

### Statistical analysis

Quantitative variables were depicted as mean with standard deviation while categorical data were described as number with percentage. The Kolmogorov-Smirnov test was performed to evaluate whether quantitative data were normally distributed. The difference in age was compared using the independent *t* test. The chi-square test was performed to compare the categorical variables of demographic features. The relationship between the biomarkers and PJI was assessed by the Mann-Whitney *U* test followed by multivariate logistic regression (forward likelihood ratio method). Covariates of operative joint, age, sex, hypertension, and diabetic status were included in the multivariate model to adjust for the confounding effects. All statistical analyses were carried out using SPSS 22.0 (IBM Corporation, Armonk, NY, USA). On the other hand, receiver operating characteristic (ROC) analysis was carried out to establish the diagnostic performance of serum proteins by MedCalc 19.0.7 (MedCalc Software, Ostend, Belgium). Several parameters, including sensitivity, specificity, area under the curve (AUC), and diagnostic odds ratio (DOR), were employed. Typically, AUC > 0.7 was considered acceptable. The optimal threshold for the diagnosis of PJI was determined by the Youden index. *P* < 0.05 was regarded as statistically significant.

## Results

### Baseline characteristics of patients undergoing revision surgery

The demographic and clinical characteristics of the enrolled patients, aseptic (*n* = 89) and septic (*n* = 38) groups, are shown in Table 1. No difference in the baseline characteristics regarding age, sex ratio, and diabetic status was observed between the two groups (*P* > 0.05). Patients in the aseptic group had a significantly higher proportion of hip issues than those in the septic group (*P* < 0.001). Interestingly, patients undergoing revision surgery for septic indication were more likely to have hypertension as opposed to the aseptic group (*P* = 0.017).

**Table 1:** The baseline characteristics of patients undergoing revision surgery

Group and variable	Aseptic revision	PJI	*P* value
No. of patients	89	38	
Age^a^	61.85 ± 13.39	63.95 ± 14.08	0.429^c^
Sex^b^			0.924^d^
Female	50 (56.2%)	21 (55.3%)	
Male	39 (43.8%)	17 (44.7%)	
Joint^b^			< 0.001^d^
Hip	79 (88.8%)	21 (55.3%)	
Knee	10 (11.2%)	17 (44.7%)	
Diabetes mellitus^b^			1.000^d^
Diabetic	11 (12.4%)	5 (13.2%)	
Nondiabetic	78 (87.6%)	33 (86.8%)	
Blood pressure^b^			0.017^d^
Hypertension	21 (23.6%)	17 (44.7%)	
Nonhypertension	68 (76.4%)	21 (55.3%)	

*P* < 0.05 was regarded as statistically significant

*PJI* periprosthetic joint infection

^a^Data are presented as the mean ± standard deviation

^b^Data are presented as the number (percentage) of patients

^c^P value was calculated by the independent t test

^d^P value was calculated by the chi-square test

### Comparisons of serum proteins between septic and aseptic groups

**Fig. 1:**
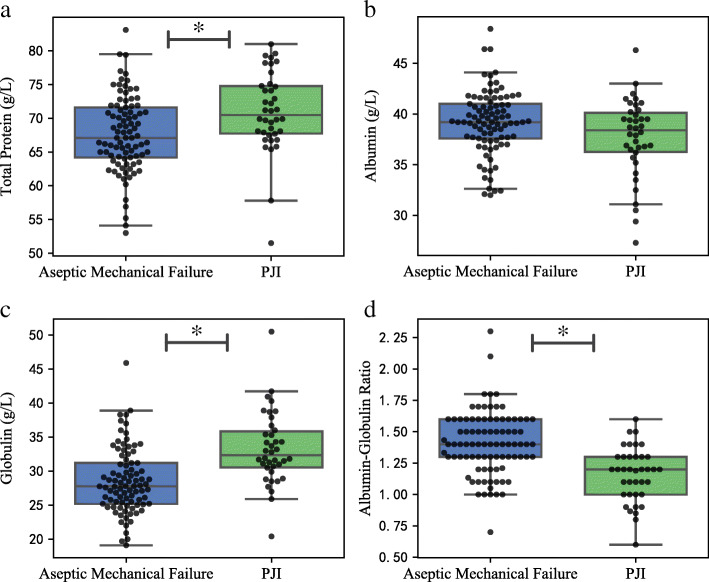
The comparison of serum proteins between patients with aseptic mechanical failure and PJI. **a** Total protein, **b** albumin, **c** globulin, and **d** albumin-globulin ratio. The asterisks indicate a significant difference between groups. The *P* values were calculated by the Mann-Whitney *U* test and adjusted by the multivariate logistic regression. *P* < 0.05 was regarded as statistically significant. Solid lines within the box indicate median, top, and bottom lines of box equal interquartile range (IQR), whiskers indicate values within 1.5 IQR of the top or bottom of the box, and dots represent outliers

The serum protein biomarkers (total protein, albumin, globulin, and A/G ratio) were evaluated between two groups using univariate analysis followed by a multivariate model. High total protein and globulin levels, as well as low A/G ratio, were significantly associated with risk of developing PJI in univariate analysis (*P* < 0.05), and the associations remained significant in a multivariate logistic regression model after adjusting for the demographic differences (OR 1.153 and *P* = 0.001 for total protein, OR 1.239 and *P* < 0.001 for globulin, OR 0.007 and *P* < 0.001 for A/G ratio, respectively). However, there was no significant difference between the two groups regarding the albumin level (*P* > 0.05) (Table 2, Fig. 1).

**Table 2:** The comparison of serum proteins between patients with aseptic and septic revision

Serum protein	Aseptic revision^a^	PJI^a^	*P* value	OR	95% CI	Adjusted *P* value
TP (g/L)	67.69 ± 5.65	70.98 ± 5.99	0.002	1.153	1.058–1.256	0.001
ALB (g/L)	39.16 ± 3.17	37.68 ± 3.91	0.055	–	–	0.156
GLB (g/L)	28.53 ± 4.76	33.30 ± 5.32	< 0.001	1.239	1.123–1.367	< 0.001
A/G ratio	1.41 ± 0.25	1.16 ± 0.22	< 0.001	0.007	0.001–0.072	< 0.001

*PJI* periprosthetic joint infection, *CI* confidence interval, *TP* total protein, *ALB* albumin, *GLB* globulin, *A/G* albumin to globulin

^a^Data are presented as the mean ± standard deviation. *P* value was calculated by the Mann-Whitney *U* test. The adjusted *P* value was assessed by the multivariate logistic regression (forward likelihood ratio method) regarding operative joint, age, sex, hypertension, and diabetic status. *P* < 0.05 was regarded as statistically significant

### The diagnostic performance of serum proteins in detecting PJI

**Fig. 2:**
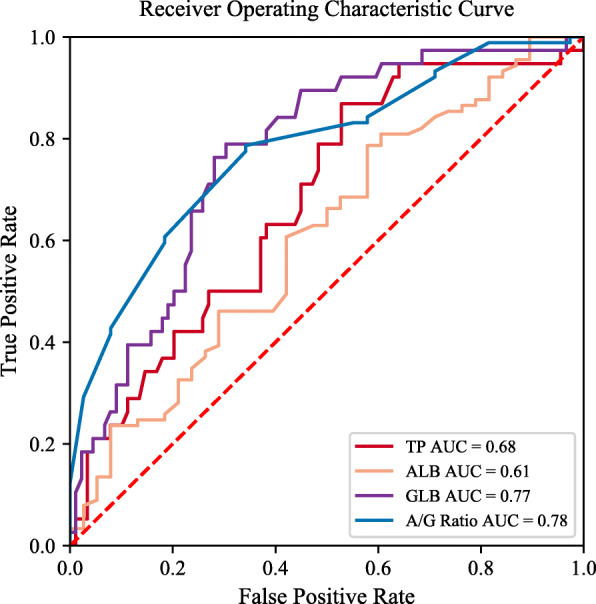
The ROC curves of serum proteins in the diagnosis of periprosthetic joint infection. TP, total protein; ALB, albumin; GLB, globulin; A/G, albumin to globulin; AUC, area under the curve

To investigate the discriminatory value of serum proteins, ROC curve analysis was performed. The AUC value of globulin (0.770, 95% confidence interval [CI] 0.687–0.840, *P* < 0.001) and A/G ratio (0.779, 95% CI 0.697–0.848, *P* < 0.001) was higher than that of total protein (0.678, 95% CI 0.589–0.758, *P* < 0.001) and albumin (0.608, 95% CI 0.517–0.693, *P* = 0.053). According to the Youden index, the optimal threshold of globulin was shown to be 29.8 g/L with a sensitivity of 78.95%, a specificity of 69.66%, and a DOR of 8.61. The optimal threshold for A/G ratio was found to be 1.2 (sensitivity 65.79%, specificity 78.65%, and DOR 7.08). The diagnostic performances of globulin and A/G ratio were considered reasonable in comparison to the total protein and albumin (Table 3, Fig. 2).

**Table 3:** The diagnostic performances of serum proteins in detecting PJI

	TP	ALB	GLB	A/G ratio
AUC	0.678	0.608	0.770	0.779
95% CI	0.589–0.758	0.517–0.693	0.687–0.840	0.697–0.848
*P* value	< 0.001	0.053	< 0.001	< 0.001
Threshold	66.4	37.3	29.8	1.2
Sensitivity (%)	86.84	42.11	78.95	65.79
Specificity (%)	47.19	78.65	69.66	78.65
PPV (%)	41.3	45.7	52.6	56.8
NPV (%)	89.4	76.1	88.6	84.3
+LR	1.64	1.97	2.60	3.08
−LR	0.28	0.74	0.30	0.43
Accuracy (%)	59.05	67.72	72.44	74.80
DOR	5.90	2.68	8.61	7.08

*PJI* periprosthetic joint infection, *TP* total protein, *ALB* albumin, *GLB* globulin, *A/G* albumin to globulin, *CI* confidence interval, *AUC* area under the curve, *PPV* positive predictive value, *NPV* negative predictive value, *+LR* positive likelihood ratio, *−LR* negative likelihood ratio, *DOR* diagnose odds ratio

## Discussion

Our findings suggest that easily accessible globulin and A/G ratio were associated with PJI and could be used as the reasonable biomarkers to diagnose PJI. Although several biomarkers related to the infection or inflammation are recommended critical in the diagnosis of PJI by MSIS guidelines, there is currently no convincible consensus available regarding the most appropriate and reliable diagnostic biomarkers. Globulin and A/G ratio represent the systemic inflammatory conditions and immune status [11–15]. However, no scientific evidence supporting their role as diagnostic markers in PJI is present.

Globulin, as a major serum protein component, is synthesized and secreted mostly by the liver and plasma cells in response to inflammatory and infective reactions. Antibodies and inflammatory cytokines are the two major components of globulin [10]. Another serum protein, albumin, has been reported to be negatively regulated by the acute phase reactant and is considered as a biomarker of inflammation and nutritional status [17, 18]. Collectively, the inverse relationship between globulin and albumin in response to inflammation and infection functions to dramatically decrease the A/G ratio, suggesting that the A/G ratio is strongly representing the inflammatory status and infection of our body [16]. Therefore, we newly introduced globulin and A/G ratio, reflected the patient’s inflammatory and infection status, as biomarkers of PJI.

No research has been studied regarding the relationship between globulin, A/G ratio, and PJI. However, various clinical studies have validated the potential role of globulin and A/G ratio in the pathogenesis of inflammatory and infectious diseases [12, 13, 19, 20]. A previous study demonstrated a strong association between high levels of serum globulin and extent of hepatic fibrosis in patients with chronic hepatitis B infection [19]. Similarly, Schmilovitz-Weiss et al. reported that high serum globulin levels could serve as a marker to predict the extent of hepatic fibrosis in patients with post-transplant recurrent hepatitis C infection [13]. Moreover, low A/G ratio is a useful marker to predict poor prognosis in cancer patients [16, 21]. In cervical cancer, high globulin levels and low A/G ratio were linked with inflammation and poor prognosis [16]. In accordance with these results, we also observed that high globulin levels and low A/G ratio were independently associated with the risk of PJI and the associations remained significant even after accounting for demographic variables. Further, ROC curve analysis revealed that globulin and A/G ratio showed acceptable predictive value for the diagnosis of PJI. With regard to the newly emerged biomarkers for PJI, our biomarkers have better diagnostic performance than platelet [22], but its related parameter, platelet to mean platelet volume ratio, was shown to be effective when combined with ESR and CRP [9]. Additionally, the diagnostic performance of D-dimer and interleukin-6 in predicting PJI was inconsistent [5, 23–27]. For example, D-dimer was reported by Qin et al. as a promising biomarker for diagnosing PJI [28] while Xu et al. demonstrated that D-dimer had limited value [24]. Nonetheless, both biomarkers have lower diagnostic performance than ESR, CRP, and plasma fibrinogen which have been reported repeatedly to have better diagnostic power (in terms of AUC, sensitivity, specificity) in the diagnosis of PJI [5, 8, 22, 23, 29]. However, the AUC of globulin and A/G ratio was larger than 0.7, indicating an acceptable diagnostic performance. In contrast, the diagnostic value of total protein and albumin was limited. Interestingly, albumin did not show significant difference between septic and aseptic groups in our study, which was inconsistent with the results of some previous studies [30, 31]. The discrepancy could be due to heterogeneity of study populations, sample sizes, and patient selection [31, 32].

Another reason for investigating the association between globulin, A/G ratio, and PJI was due to the observation that patients who underwent TJA were more likely to be elderly and exhibited comorbidities such as diabetes mellitus and renal insufficiency, all of which may be associated with poor immunity and higher complications, including PJI [31]. As the preoperative globulin and A/G ratio are crucial parameters to evaluate the general and immune status of the patients, any abnormal changes in these two biomarkers should be further investigated. Although not proven by the present study, we hypothesize that evaluation of patients with higher globulin or low A/G ratio and correction of these parameters might prevent postoperative complications. Besides, all the patients who plan to have surgery are required to screen the basic metabolic panel which contains globulin and A/G ratio. Hence, globulin and the A/G ratio are cost-effective and easily accessible parameters, in which patients do not need to get more tests checked preoperatively.

To the best of our knowledge, this is the first instance to illustrate the association of globulin and A/G ratio with PJI. However, the current study is not without limitations. First, the study was not able to include all the confounding variables, for example, invisible inflammatory conditions or infections, which might impact the globulin levels. Nevertheless, we did exclude some of the common inflammatory and infective diseases (e.g., tuberculosis). And multivariate logistic regression analysis was performed to avoid the unwanted impact of confounding factors. Second, the current study was a retrospective review and we were unable to determine the causality and direction of the relationship between globulin, A/G ratio, and PJI. Despite that, our study did reveal that there was a strong association between globulin, A/G ratio, and PJI. Third, globulin includes four categories of proteins. In our study, we did not separate globulin into specific molecules by electrophoresis because of the retrospective design of the study. However, we hope that future research could pay attention to the association between the four categories of globulin and PJI. Fourth, this was a single-center study and a relatively small number of patients were recruited, particularly in the septic group. Large multicenter studies are required to validate the present findings. Nevertheless, data from one center with controlled laboratory protocols strengthen our study’s methodology as this could be more generalized and decrease the heterogeneity between groups. Finally, our study cohort included patients requiring revision arthroplasty, which could miss patients with asymptomatic infections or mild clinical manifestations.

## Conclusions

Our study elucidated that the easily accessible globulin and A/G ratio were significantly associated with PJI and had the potential to be used as diagnostic biomarkers. Future robust studies implementing standardized protocols in a large-scale cohort should be performed to establish the use of these two biomarkers in the diagnosis of PJI, and subsequent correction of abnormal biomarker values is warranted.

## Data Availability

The datasets used during the current study are available from the corresponding author on reasonable request.

## Abbreviations

TJA: Total joint arthroplasty
PJI: Periprosthetic joint infection
MSIS: Musculoskeletal infection society
TP: Total protein
ALB: Albumin
GLB: Globulin
A/G: Albumin to globulin
CRP: C-reactive protein
ESR: Erythrocyte sedimentation rate
ROC: Receiver operating characteristic
AUC: Area under the curve
PPV: Positive predictive value
NPV: Negative predictive value
+LR: Positive likelihood ratio
−LR: Negative likelihood ratio
DOR: Diagnostic odds ratio
SD: Standard deviation
